# Phase 1 trial of same day cytology to guide the use of HIPEC

**DOI:** 10.1097/SP9.0000000000000017

**Published:** 2024-01-20

**Authors:** Paul H. Sugarbaker, Tom Deng

**Affiliations:** a Program in Peritoneal Surface Malignancy, Washington Cancer Institute; b Department of Pathology, MedStar Washington Hospital Center, Washington, DC, USA

**Keywords:** colorectal cancer, gastric cancer, hyperthermia, mucinous appendiceal neoplasms, ovarian cancer, peritoneal cytology, pseudomyxoma peritonei, peritoneal metastases, tumor cell entrapment

## Abstract

**Background:**

Peritoneal metastases from gastrointestinal or gynecologic malignancy are a prominent part of the natural history of these diseases. Peritoneal metastases, if not effectively treated, will result in a decreased survival and cause an impaired quality of life. Hyperthermic intraperitoneal chemotherapy (HIPEC) is a treatment specifically designed to combat peritoneal metastases. A group of patients who, from a theoretical perspective, may benefit from HIPEC are those patients with a positive peritoneal cytology. In order to identify these patients at the time of a surgical intervention, a same day cytology is to be performed.

**Materials and methods:**

The result of this test is to be available at or before the completion of the cancer resection. If the cytology is positive, the patient immediately becomes a candidate for HIPEC. The HIPEC will be of maximal value if a complete cytoreduction, as judged by the surgeon, has been possible. This phase 1 trial is to demonstrate that the Surgical Oncology Service, the Department of Pathology, the Pharmacy and the Operating Room personnel can co-ordinate a phase 1 protocol to successfully complete the same day cytology with an efficient delivery of HIPEC. A standardized plan for consent, cytology collection, preparation of the specimen, reading of the specimen, reporting the results in a timely manner facilitates the administration of HIPEC in peritoneal cytology positive patients.

**Dissemination:**

Successful completion of these requirements is a positive result for this study and allows for future protocols to be generated. Successful completion of the same day cytology phase 1 protocol will allow the efficacy, safety, and efficiency of this plan of patient management to be evaluated.

Highlights

- The determination of positive versus negative peritoneal cytology at the time of a surgical intervention is not currently routinely performed.
- The methodology for making the results of peritoneal cytology available in the operating theater is not currently in use.
- A standardized use of hyperthermic intraperitoneal chemotherapy (HIPEC) is in patients with positive peritoneal cytology.
- A workable protocol to identify positive peritoneal cytology during a surgical procedure as an indication for HIPEC is a necessary first step toward improved patient care.
- Optimal management plans for patients found to have positive peritoneal cytology at the time of a surgical procedure need to be determined.

## Background and rationale

### Credits and debits regarding the use of HIPEC

Peritoneal metastases are a prominent part of the natural history of many abdominal and pelvic malignancies. Elimination of this site for metastatic disease may improve survival and, if not, improve survival have a positive impact on quality of life. Quality of life improvements would come about by prolongation of gastrointestinal function.

From a pharmacologic perspective, administration of cancer chemotherapy directly into the peritoneal cavity results in a regional dose intensity that can be exploited for greater cytotoxicity^1^. Hyperthermia within the peritoneal cavity to 42°C can augment this dose intensity. These positive aspects of intraperitoneal chemotherapy are diminished by the limited penetration of drugs of only a few cell layers into cancer nodules or the peritoneal surface contaminated by cancer cells. This limited penetration occurs because it is driven by simple diffusion. A high concentration of cancer chemotherapy within the chemotherapy solution causes a movement of drug into the low concentration within tumor nodules. If the tumor nodule is vascularized, the cancer chemotherapy will be rapidly moved into the body compartment. If the high concentration of chemotherapy is removed from the peritoneal space, it may be rapidly eluted from the cancer nodule.

### Delay in the administration of HIPEC as a cause of treatment failure

If cancer cells are placed into the peritoneal cavity of an experimental animal, intraperitoneal chemotherapy can be used effectively to control the progression of peritoneal metastases. However, this regional chemotherapy treatment is not effective if the intraperitoneal administration of chemotherapy is delayed. Simultaneous inoculation of the cancer cell suspension and cancer chemotherapy is the most effective. After a 1 h delay, effects of treatment are reduced. After 24 h, the benefits of intraperitoneal chemotherapy are gone^2^.

In current practice, peritoneal cytology is often sent at the beginning and sometimes at the end of a cancer resection. The results are available within 3–4 days because the cell block is not available until this time. These delayed results provide valuable information regarding staging and prognosis. However, they are of no therapeutic benefit. The free intraperitoneal cancer cells have become tightly adherent to resection sites and traumatized peritoneal surfaces. They are trapped within a matrix of organized fibrin and are no longer accessible to intraperitoneal chemotherapy.

In contrast, same day cytology would give the oncologic surgeon information that could be used therapeutically. It would allow HIPEC to be used as part of a cancer resection. The patients most likely to benefit from HIPEC would be identified and available for treatment with optimal timing. An efficient and cost-effective execution of same day cytology is the goal of this protocol.

### Treatment of cancer cells loosely adherent to surfaces of the abdomen and pelvis

These debits of intraperitoneal chemotherapy delivery are avoided and credits maintained if the target for the intraperitoneal chemotherapy is single cancer cells within the peritoneal fluid. This type of cancer spread is identified by a positive peritoneal cytology. A second target that can be treated by intraperitoneal chemotherapy is cancer cells disrupted as a result of surgical trauma that have become loosely adherent to parietal or visceral peritoneal surfaces^3^. These cancer cells accumulate on raw tissue surfaces, especially at the sites of cancer resection. They are single cells that can be dislodged from the traumatized tissue surfaces by large volumes of intraperitoneal chemotherapy solution. To eradicate free intraperitoneal cancer cells and cancer cells loosely adherent to abdominal and pelvic surfaces, the warm chemotherapy solution must flood the peritoneal cavity with a relative uniform distribution. For best results, this peritoneal lavage must occur soon after the complete resection of the primary cancer and the complete resection of any tumor nodules that are securely attached to peritoneal surfaces. Delay in the delivery of the intraperitoneal chemotherapy solution will allow the cancer cells to be trapped within an organized fibrinous layer that will occur within a few hours as part of the healing process.

### Increasing the viable targets for HIPEC with same day cytology

The minimal or absent HIPEC effects demonstrated to date result from HIPEC application in clinical situations in which HIPEC has little or no efficacy^4^. A plan to use HIPEC only when the heated chemotherapy solution has free cancer cells as a target is possible with the initiation of a program in ‘Same Day Cytology’. This clinical tool would provide information to the surgical oncologist regarding the presence of free intraperitoneal tumor cells. These cells identified by positive cytology are the most efficient target of HIPEC. If the same day cytology report returns positive, an appropriate HIPEC for a particular disease should be administered. There may be some clinical situations in which HIPEC may be indicated even if the same day cytology is negative. For example, if a very extensive resection of an advanced gastric cancer is required, the likelihood of cancer cells released as a result of surgical trauma is large. Information regarding peritoneal cytology as positive after gastrectomy is not likely to be available to the surgeon in a timely manner to decide if HIPEC is indicated. In some clinical situations, the HIPEC should be administered in the absence of information regarding positive cytology.

### Low-grade appendiceal mucinous neoplasm with peritoneal metastases – possible benefits of HIPEC

The low-grade appendiceal mucinous neoplasms cause extensive accumulation of mucus and numerous strips of minimally atypical epithelial cells. These cells are not invasive into the peritoneum so they are easily dislodged from a surface to which they are adherent. The HIPEC can destroy the large number of cells that remain after cytoreduction within the free peritoneal space and loosely adherent to structures within the abdomen and pelvis. The mucus that surrounds each cell may act as a reservoir for chemotherapy solution and thereby increase the residence time of the chemotherapy. Cytoreductive surgery plus HIPEC is accepted as a standard of care for low-grade appendiceal mucinous neoplasms^5^.

### Colorectal cancer peritoneal metastases – possible benefits from HIPEC

A positive impact on the occurrence of local-regional disease has been demonstrated in T4 colon cancer^6^. As expected, HIPEC mitomycin C was effective in controlling free cancer cells present within the peritoneal spaces if gross disease was not present at other sites. In contrast, HIPEC is expected to be less effective in treating established peritoneal metastases. In patients with peritoneal metastases from colon cancer only 50% of patients will have a positive cytology^7^. Many patients, in the absence of free cancer cells, will not have a target for HIPEC. A single cycle of HIPEC for metachronous peritoneal metastases can only be expected to result in benefit for a small proportion of patients.

### Gastric cancer peritoneal metastases – possible benefits from HIPEC

Gastric cancer with peritoneal metastases has a high incidence of peritoneal metastases and positive peritoneal cytology. The chemotherapy agents used for gastric cancer HIPEC are usually effective and the destruction of free cancer cells or cells loosely adherent to peritoneal surfaces is expected. Control of free cancer cells and their elimination from the resection site and other abdominal and pelvic surfaces will result in an absence of peritoneal metastases that would progress in the future^8^. Larger nodules, vascularized nodules or cancer cells caught up in scar will not be eliminated.

### Ovarian cancer – possible benefits from HIPEC

Ovarian cancer with peritoneal seeding has clinical features that are likely to be benefitted by HIPEC. First, in a large majority of patients free intraperitoneal cancer cells are present as positive cytology. In addition, after the cytoreductive surgery to resect the ovarian cancer the abdominal and pelvic surfaces are contaminated by cancer cells spread around the peritoneal cavity as a result of the trauma of surgery. These loosely adherent cells are targets for an effective HIPEC. As a result of this eradication of cancer cells by HIPEC at the time of surgery, scar tissue infiltrated by tumor can be avoided. The layer of cancer-contaminated scar can be prevented by HIPEC. Tumor cells trapped in scar tissue are an ineffective target for HIPEC. Also, tumor cells trapped in scar are not an effective target for systemic chemotherapy. Elimination of tumor cell entrapment in scar would cause HIPEC to improve the outcome of patients with ovarian cancer. The systemic chemotherapy with cisplatin and paclitaxel is expected to be effective at systemic sites but minimally effective for poorly vascularized tumor trapped within scar tissue^9^.

### Endometrial cancer peritoneal cytology – possible benefits from HIPEC

The predominant anatomic sites for endometrial cancer dissemination are not the peritoneal surfaces. Only ~10% of patients with disease confined to the uterus have a positive peritoneal cytology. These patients have a reduced prognosis and the recurrence of disease is, in a majority of cytology positive patients, as peritoneal metastases^10^. Same day cytology would identify these patients and reduce, probably eliminate, progression of peritoneal metastases in this special group of patients. Any improvement in long-term survival would need to be established with a randomized trial.

### Incidence of free cancer cells in the absence of gross peritoneal metastases in gastrointestinal and gynecologic cancers

Rekhraj and colleagues performed a meta-analysis to determine the impact of free intraperitoneal cancer cells on survival of primary colon cancer resections. They reported on nine studies with a total of 1182 patients. Free cancer cells were detected prior to resection in 125/822 (15.2%) of patients and in 64/533 (12%) of patients following resection. Positive peritoneal cytology was associated with a significantly higher incidence of overall recurrence in both preresection and postresection groups^11^.

In patients with gastric cancer, a preoperative and postoperative peritoneal cytology has become a standard of care and is an important independent prognostic indicator. Positive peritoneal cytology moves a patient into the stage pM1 pathologic classification according to the Eighth Edition of the American Joint Cancer Staging Manual^12^. At Memorial Sloan-Kettering Cancer Center, 1241 patients had laparoscopy preoperatively as staging for gastric cancer. They report 93 patients (32%) without gross evidence of advanced disease (no peritoneal metastases detected) with positive peritoneal cytology. They conclude that patients with positive peritoneal cytology as the only evidence of advanced disease exhibiting a poor outcome^13^.

For ovarian cancer, the incidence of visible peritoneal metastases approaches 100%. Peritoneal cytology is used to confirm the diagnosis but would not be the indication for a HIPEC procedure. Peritoneal cytology is suggested to be of value in women with uterine leiomyosarcoma. Matsuo and coworkers reported on 1481 uterine sarcomas. Malignant peritoneal cytology was present in 146 patients (9.9%)^14^. Also, positive peritoneal cytology was reported as an independent risk factor in patients with early stage endometrial cancer^15^.

In summary, peritoneal cytology is often positive in the absence of visible peritoneal metastases or other evidence of disseminated disease. The present of peritoneal metastases has a profound impact on prognosis. Survival may be improved if HIPEC is used in a timely manner on this subset of patients.

### Summary regarding the use of HIPEC

The use of HIPEC in gastrointestinal and gynecologic malignancy has been used in many clinical situations that were unlikely to result in benefit. Only single cells suspended within the chemotherapy solution are reliably destroyed by a single HIPEC treatment. Visible nodules even less than 1 mm, vascularized nodules or plaques, and tumor cells layered out in scar tissue are not an effective target for HIPEC. Destruction of free intraperitoneal tumor cells and prevention of cancer from being entrapped in scar tissue can eliminate a major site for systemic chemotherapy failure. In order to confirm that patients with a high likelihood of benefit from HIPEC do exist, the routine application of same day cytology is recommended.

### Goals of this protocol

The aim of our study is to show that HIPEC can be used as a planned part of a resection of an abdominal or pelvic malignancy in order to eradicate free intraperitoneal cancer cells and cancer cells loosely adherent to traumatized abdominal or pelvic surfaces. To this point in time, identification of the patients who will benefit from HIPEC has not been definitively established. The hypothesis of our study is that a positive peritoneal cytology identifies a patient who, along with a complete resection of visible disease, will benefit from HIPEC. In the current phase 1 study, the results of the peritoneal cytology can be made available with 3–4 h. A positive result causes the patient to be a candidate for HIPEC. Positive results of the same day cytology are used as an indication for HIPEC if the cancer resection is complete.

## Methods/design

### Objectives

The goal of this study is to demonstrate the feasibility of a same day cytology to select patients with a complete cancer resection for HIPEC. In order for this strategy to be successful the Surgical Oncology Service, the Department of Pathology/Cytology, the Hospital Pharmacy, and the Operating Room personnel must provide a coordinated effort.

### Methods

**Figure 1:**
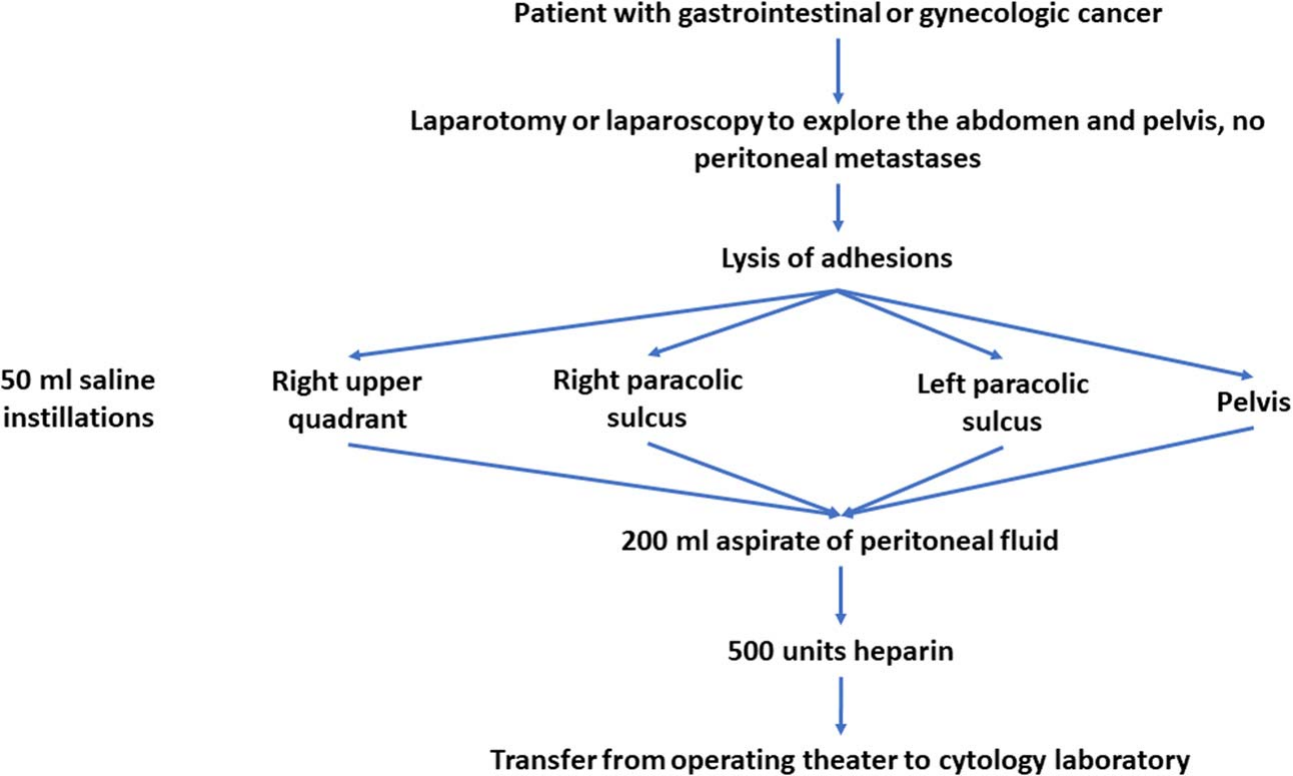
Flow chart for obtaining 200 ml of peritoneal fluid.

1. A first requirement is preoperative consent for HIPEC in gastrointestinal and gynecologic malignancy patients having abdominal-pelvic surgery for cancer (Tables 1, 2). Patients with a primary or recurrent cancer will require not only a standard consent for complete resection of the malignancy but also consent for HIPEC.
2. The surgical procedure scheduled with the operating room must also include collection of the same day cytology specimen and possible administration of HIPEC. In order to avoid unnecessary delay, the equipment required for same day cytology must be available prior to the start of the surgical procedure. Also, the administration of the standard HIPEC for gastrointestinal or gynecologic malignancy must be possible with a few minutes notice.
3. Collection of the same day cytology specimens: The same day cytology specimen is to be collected just after access to the abdomen and pelvis has been obtained (Fig. 1). Adhesions should be lysed to gain visual access to the right upper quadrant, right paracolic sulcus, left paracolic sulcus, and deep pelvis. An exploration of the abdomen and pelvis with a full description of the findings should be recorded in the operative note dictated by the surgeon. Prior to collection of the same day cytology specimen lysis of adhesions or surgical dissection that results in bleeding must be avoided. A bloody same day cytology specimen is more difficult to read. Also, blood contaminates the peritoneal fluid and will dilute the number of malignant cells identified.
  Four specimens of 50 ml each are to be obtained from each patient. If the patient has ascites no saline irrigation is required. Forcibly instill 50 ml into the right upper quadrant (superior and inferior to the liver), into the right paracolic sulcus, into the left paracolic sulcus, and into the deep pelvis posterior to the rectum. If the peritoneal fluid collection from each anatomic site is not equal to 50 ml, instill the volume of saline needed to provide a 50 ml specimen. The aspirations each containing 50 ml, should be contained in a suction container dedicated to the recovery of 200 ml of peritoneal fluid aspirate. It should leave the operating room table and be handed off to go to the cytology laboratory (800 cc Suction Canister, Cardinal Health Inc.).
  One of the assumptions of this phase 1 study is that a lavage of four anatomic sites at the time of the exploration of the abdomen and pelvis in a surgical procedure produces a higher likelihood of a positive result that a paracentesis. If same day cytology is available, it can simplify patient management and reduce the incidence of false negative peritoneal cytology.
4. Preparation of the same day cytology specimen for the pathologist/cytologist: The 200 ml suction container is handed off the operating table, immediately uncapped and 500 units of heparin added. The 200 ml suction container is swirled to disperse the heparin. The 200 ml of peritoneal irrigation fluid or ascites are transferred to the Pathology/Cytology Department. Tubes containing the peritoneal fluid are centrifuged at 2000 rpm for 10 min. The fluid is suctioned off taking care to preserve the cellular pellet at the base of each tube. The cellular pellets from three tubes are agitated by pipette and transferred to the fourth tube. This tube is recapped, centrifuged, and transferred to the pathologist/cytologist for staining and reading.
5. Staining and reading of the same day cytology specimen: A portion of the sediment is smeared on four glass slides. Two slides should be smeared with ~1 ml of sediment and prepared for microscope study by the Papanicolaou thin prep method. Two slides should be smeared with ~1 ml of sediment and allowed to air dry. They will be prepared for microscope study by the Diff Quick method. The remainder of the cellular pellet is submitted for cell block. Hematoxylin and eosin stain and appropriate immunostains for the malignancy being treated will supplement the same day cytology report several days later.
6. Interpretation of the slides for same day cytology: A slide should be interpreted as positive if clusters of or individual cells are detected. For an experienced cytopathologist differences between cancer cells and reactive mesothelial cells should be apparent.
7. Time allowed for the final report for the same day cytology: The primary goal of this pilot study is to demonstrate the feasibility and reproducibility of the same day cytology. To be successful the results of the cytology must be available when the cancer specimen is removed. A 3 h time allowed for the final report of the same day cytology is required. Early reporting is of benefit in that preparation for HIPEC will be facilitated.
8. Standard HIPEC for positive same day cytology: If the same day cytology is positive a standardized HIPEC regimen is performed. The HIPEC methodology may be open or closed at the discretion of the surgical oncology team. The chemotherapy agents to be used are selected by the peritoneal surface malignancy team. The HIPEC treatment is maintained for 90 min at 42°C in the whole abdomen and pelvis.
9. Review of slides obtained by same day cytology: All slides will be stored for consultation within a group meeting of pathologists/cytologists from all participating institutions. A record of positive cytologies by digital photography is recommended. All negative slides will be reviewed to assess the quality of the staining technology.
10. Accrual: The plan is to continue the study until 20 positive same day cytology reports with HIPEC administered have been achieved. The total number of patients in the feasibility trial cannot be estimated at this time.
11. Future plans: A randomized trial to test the efficacy of HIPEC in patients with positive same day cytology is planned.
12. Ethics approval and consent to participate from patients for this study will be obtained.

**Table 1:** Subject inclusion criteria.

• Patient age 18 years or older, both sexes. • Clinical diagnosis of primary gastrointestinal or gynecologic malignancy. • Patient must be planning to undergo complete resection of all primary tumor. • ECOG performance status of less than or equal to 1. • Hematology: ANC greater than or equal to 1500/µl. • Platelets: greater than 75 000/µl. • Adequate renal function: creatinine less than 1.5× the upper limit of normal (ULN) or calculated creatinine clearance of greater than or equal to 50 ml/min. • Adequate hepatic function: bilirubin less than 1.5 mg/dl (except in patients with Gilbert’s syndrome, who must have a total bilirubin <3.0 mg/dl). • Women of childbearing potential with a negative pregnancy test result (urine or blood) who agree to use an effective contraceptive method. Reliable contraception should be used from trial screening and must be continued throughout the study. A woman of childbearing potential is defined as one who is biologically capable of becoming pregnant. • A man participating in this study must agree to utilize a reliable barrier form of contraception for the duration of the study. • Signed and dated written informed consent to participate in this clinical trial must be obtained prior to any study procedure.

**Table 2:** Subject exclusion criteria.

• Subjects who have previously undergone intraperitoneal chemotherapy. • Subjects with preoperative or intraoperative biopsy consistent with peritoneal metastases or disseminated disease. • Other prior malignancies, except for cured non-melanoma skin cancer, curatively treated *in situ* carcinoma of the cervix, adequately treated malignancies for which there has been no evidence of activity for more than three years, or indolent tumors for which observation over 2 years is a reasonable option. • High suspicion for extra-abdominal metastases. • Women who are pregnant or lactating. • Subjects with a condition which may interfere with their ability to understand the requirements of the study. • Active coronary artery disease (defined as unstable angina or a positive cardiac stress test). Subjects with a history of coronary artery disease may be included if they have had a normal stress test within 60 days of enrollment or are determined by a cardiologist to be of acceptable perioperative risk. • Uncontrolled hypertension defined as greater than 140/90 and not cleared for surgery at the time of consent. • New York Heart Association (NYHA) Class II or higher congestive heart failure; restrictive or obstructive pulmonary disease that would limit study compliance or place the patient at unacceptable risk for participation in the study. • History of cerebrovascular disease that would limit study compliance or place the patient at unacceptable risk for participation in the study. • Subjects with other concurrent severe medical problems unrelated to the malignancy that would significantly limit full compliance with the study or place them at unacceptable risk for participation in the study. • Patients with known cisplatin, carboplatin, or pemetrexed allergy. • Evidence of extensive intraperitoneal adhesions at the time of surgery, which prohibits intraperitoneal therapy, as determined by the operating surgeon. • Any condition that would preclude the ability to deliver appropriate intraperitoneal chemotherapy. • Use of an oral medication, lacking a suitable nonoral substitute, that if held for up to 10 days would be felt an unacceptable risk by the investigator. • Life expectancy less than 12 weeks.

## Ethical approval and consent to participate

The phase I protocol is to be offered to a single as yet not identified institution. This institution will construct an appropriate consent with the ethics committee/institutional review board. All cytology specimens/human data will be performed in accordance with relevant guidelines and regulations.

## Consent

The participating institution will construct an appropriate consent with the guidance of its ethics committee/institutional review board.

## Sources of funding

Administrative and secretarial support was provided by Foundation for Applied Research in Gastrointestinal Oncology (FARGO).

## Author contributions

All authors have accepted responsibility for the entire content of this manuscript and approved its submission. PHS and TD conceived and designed the study protocol. They both contributed to the intellectual content and approved the final version for publication.

## Conflits of interests disclosure

Authors state no conflicts of interest.

## Research registration unique identifying number (UIN)

Not applicable.

## Guarantor

Paul H. Sugarbaker.

## Data availability statement

Data sharing not applicable to this article as no datasets were generated or analyzed during the writing of the current study protocol.

## Provenance and peer review

Not an invited paper.
